# [Retracted] lncRNA‑u50535 promotes the progression of lung cancer by activating CCL20/ERK signaling

**DOI:** 10.3892/or.2024.8785

**Published:** 2024-07-29

**Authors:** Wei Wei, Xiaoliang Zhao, Jianquan Zhu, Lianmin Zhang, Yulong Chen, Bin Zhang, Yue Li, Meng Wang, Zhenfa Zhang, Changli Wang

Oncol Rep 42: 1946–1956, 2019; DOI: 10.3892/or.2019.7302

Following the publication of this paper, it was drawn to the Editor's attention by a concerned reader that certain of the western blotting data shown in Fig. 4B and C on p. 1952, and the Transwell invasion assay data in Fig. 2F and 4I, had already appeared in previously published articles written by different authors at different research institutes (a number of which have been retracted). Owing to the fact that the contentious data in the above article had already been published prior to its submission to *Oncology Reports*, the Editor has decided that this paper should be retracted from the Journal. The authors were asked for an explanation to account for these concerns, but the Editorial Office did not receive a reply. The Editor apologizes to the readership for any inconvenience caused.

